# Decitabine in combination with low-dose cytarabine, aclarubicin and G-CSF tends to improve prognosis in elderly patients with high-risk AML

**DOI:** 10.18632/aging.102973

**Published:** 2020-04-01

**Authors:** Ming Hong, Han Zhu, Qian Sun, Yu Zhu, Yi Miao, Hui Yang, Hai-Rong Qiu, Jian-Yong Li, Si-Xuan Qian

**Affiliations:** 1 Department of Hematology, The First Affiliated Hospital of Nanjing Medical University, Jiangsu Province Hospital, Nanjing 210029, Jiangsu Province, China; 2 Key Laboratory of Hematology of Nanjing Medical University, Nanjing 210029, Jiangsu Province, China; 3 The Collaborative Innovation Center for Cancer Personalized Medicine, Nanjing 210029, Jiangsu Province, China

**Keywords:** acute myeloid leukemia, elderly, D-CAG, high-risk

## Abstract

We evaluated the risk status and survival outcomes of 125 elderly acute myeloid leukemia (AML) patients treated with decitabine in combination with low-dose cytarabine, aclarubicin, and G-CSF (D-CAG). The risk status was evaluated by determining the frequency of recurring gene mutations using next-generation sequencing (NGS) analysis of 23 selected genes and cytogenetic profiling of bone marrow samples at diagnosis. After a median follow-up of 12 months (range: 2-82 months), 86 patients (68.8%) had achieved complete remission after one cycle of induction, and 94 patients (75.2%) had achieved it after two cycles. The median overall survival (OS) and disease-free survival (DFS) were 16 and 12 months, respectively. In 21 AML patients aged above 75 years, the median OS and DFS were longer in the low- and intermediate-risk group than the high-risk group, but the differences were not statistically significant. The median OS and DFS were similar in patients with or without *TET2*, *DNMT3A*, *IDH2, TP53* and *FLT3* mutations. Multivariate analysis showed that patient age above 75 years, high-risk status, and genetic anomalies, like deletions in chromosomes 5 and/or 7, were significant variables in predicting OS. D-CAG regimen tends to improve the prognosis of a subgroup of elderly patients with high-risk AML.

## INTRODUCTION

Acute myeloid leukemia (AML) is a hematopoietic malignancy that affects all age groups, but predominantly occurs in the elderly with a median age of 65 years [1]. The prognosis of older patients is worse than younger patients because of higher degree of resistance to conventional chemotherapy, increased frequency of genetic mutations, decreased performance status (PS), comorbid conditions, and limited treatment options [2–5]. The complete remission (CR) rates for AML patients above 60 years that have undergone anthracycline-based induction chemotherapy ranges from 39% to 63%, but, the median overall survival (OS) and disease-free survival (DFS) remained poor (7-12 months) [6]. Furthermore, the median OS for patients above 65 years receiving anti-leukemia therapy was 6 months and the 5-year survival rate was below 5% after initial diagnosis [7].

Consequently, treating elderly AML patients is challenging. Previously, we conducted a phase II clinical study in elderly patients with the characteristic regimen of decitabine in combination with modified CAG (D-CAG), and obtained an objective response rate (ORR) of 82.4% and a CR rate of 64.7%; the median OS for patients aged ≥70 years and 60-69 years was 10 and 12 months, respectively [8]. Moreover, we demonstrated that D-CAG was as effective as the standard induction and intensive consolidation regimen for AML patients aged 55–69 years [9].

Some frequently mutated genes in AML have been identified as predictors of treatment response because of recent advances in next-generation sequencing (NGS) and the availability of AML-focused gene panels [10]. Risk stratification models based on *NPM1*, *CEBPA*, *FLT3-ITD*, *RUNX1*, *ASXL1*, and *TP5*3 mutations have been developed to predict survival and treatment response based on extensive studies in younger patients undergoing intensive chemotherapy [11]. NGS has improved our understanding of the molecular genetics underlying AML, and several gene mutations have been incorporated into existing prognostic models to precisely determine the risk status of AML patients.

In this study, we investigated the relationship between risk status, pre-treatment gene mutations, and survival outcomes of treatment with the D-CAG regimen in elderly AML patients.

## RESULTS

### Patient characteristics

The baseline characteristics of AML patients analyzed in this study are shown in Table 1. The study cohort consisted of 42 (33.6%) cases of AML with recurrent cytogenetic abnormalities (AML-RCA), 20 (16%) cases of AML with myelodysplasia-related changes (AML-MRC), nine (7.2%) cases of therapy-related AML (t-AML), and 54 (43.2%) cases of AML, not otherwise specified (AML, NOS). Cytogenetic examination revealed normal cytogenetics in 69 patients and complex karyotypes in 19 patients. The patients were classified into low-risk (12 patients; 9.6%), intermediate-risk (50 patients; 40.0%), and high-risk (63 patients; 50.4%) categories based on their cytogenetic characteristics and molecular abnormalities that were identified using NGS analysis of BM samples collected at diagnosis prior to any treatment.

**Table 1:** Patient demographic and baseline characteristics at diagnosis.

**Characteristic**	
n	125
Age, median (range)	66.0 (60-86)
Age ≥ 70, n (%)	41 (32.8)
Gender, n (%)	
Male	67 (53.6)
Female	58 (46.4)
Prior diagnosis of MDS, n (%)	8 (6.4)
WHO 2016 AML classification, n (%)	
AML with recurrent genetic abnormalities	42 (33.6)
AML with myelodysplasia-related changes	20 (16)
Therapy-related myeloid neoplasms	9 (7.2)
AML not otherwise specified	54 (43.2)
ECOG performance status score, n (%)	
0-1	106 (84.8)
2-3	19 (15.2)
BM blasts (%), median (range)	64.0 (20-90.8)
NCCN risk stratification, n (%)	
Low-risk	12 (9.6)
Intermediate-risk	50 (40)
High-risk	63 (50.4)
WBC (×10^9^/L), median (range)	11.7 (0.5-239)
Hemoglobin (g/L), median (range)	79 (46-137)
Platelets (×10^9^/L), median (range)	72.0 (6-257)

Abbreviations: ANC: absolute neutrophil count; BM: bone marrow; ECOG PS: Eastern Cooperative Oncology Group performance status; MDS: myelodysplastic syndrome; WBC: white blood cell count; WHO: World Health Organization.

### Mutational analyses

**Figure 1:**
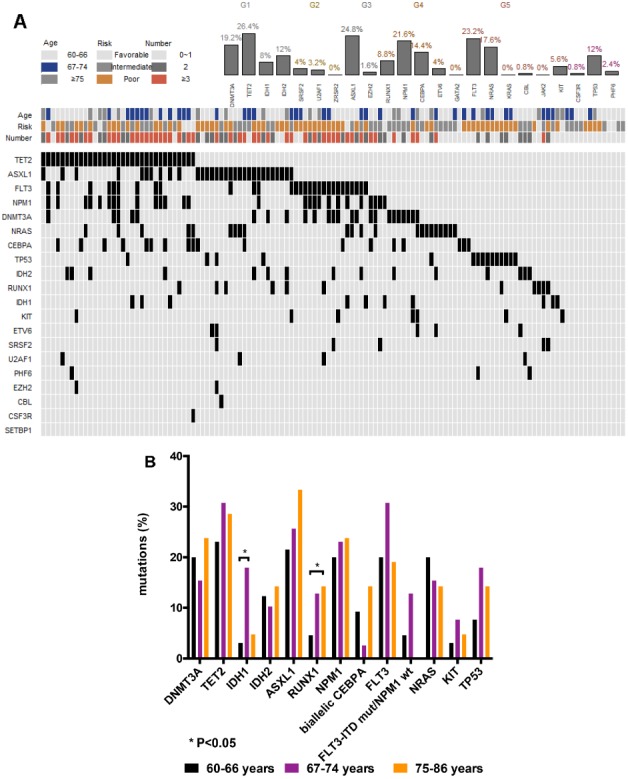
**Genetic landscape of elderly AML patients.** (**A**) Heatmap showing associations between different gene mutations. Each column represents one patient. (**B**) Gene mutations in 125 AML patients ≥ 60 years of age at primary diagnosis. Bar chart showing the 13 most commonly mutated genes in elderly AML patients aged 60-66, 67-74, and 75-86 years at primary diagnosis.

NGS analysis revealed mutations in 19 different genes in 115 (92%) patients. Among these 115 patients, 34 (27.2%) had mutations in a single gene, 35 (28%) had mutations in two genes, 28 (22.4%) had mutations in three genes, 17 (13.6%) had mutations in four genes, and one had mutations in six genes. The most frequently mutated gene was *TET2* (26.4%). The remaining mutated genes in the order of decreasing frequency were: *ASXL1*, 24.8%; *FLT3*, 23.2%; *NPM1*, 21.6%; *DNMT3A*, 19.2%; *NRAS*, 17.6%; *CEBPA*, 14.4%; *IDH2*, 12%; *TP53*, 12%; *RUNX1*, 8.8%; *IDH1*, 8.0%; and *KIT*, 5.6%; mutations in *ETV6*, *SRSF2*, *U2AF1*, *PHF6*, *EZH2*, *CBL* and *CSF3R* were rare (<5.0%); mutations were not identified in *KRAS*, *JAK2*, *ZRSR2* and *GATA2* genes (Figure 1A, Table 2). In comparison to patients with complex karyotypes, mutations in *DNMT3A* (21.7% vs. 0, P= 0.0341), *TET2* (34.8% vs. 10.5%, P= 0.0485), *NPM1* (14.5% vs. 0, P= 0.0047) and *FLT3* (23.2% vs. 0, P= 0.0184) were more common in patients with normal karyotype. Conversely, the rate of *TP53* mutations was significantly higher in patients with complex karyotypes than those with the normal karyotype (57.9% vs. 2.9%, P< 0.0001; Table 2). Ten out of fifteen patients (66.7%) with *TP53* mutations belonged to the t-AML or the AML-MRC group. We divided the patients based on their age into three subgroups (60-66, 67-74 and 75-86 years) using ROC curve analysis. Mutations in *IDH1* were more common in patients aged 67–74 years than those aged 60–66 years (18.0% vs. 3.1%, P=0.0248; Figure 1B). The rate of *RUNX1* mutations were significantly higher in patients aged 75–86 years than those aged 60-66 year age group (14.3% vs. 4.6%, P= 0.0264; Figure 1B).

**Table 2:** Mutations.

	**Mutations**	**n (%)**	**Normal cytogenetic (n=69) n (%)**	**Complex karyotypes (n=19) n (%)**	**P**
DNA methylation	*DNMT3A*	24 (19.2)	15 (21.7)	0	**0.0341**
	*TET2*	33 (26.4)	24 (34.8)	2 (10.5)	**0.0485**
	*IDH1*	10 (8)	6 (8.7)	0	0.3332
	*IDH2*	15 (12)	12 (17.4)	0	0.0619
RNA splicing	*SRSF2*	5 (4)	0	0	
	*U2AF1*	4 (3.2)	0	0	
	*ZRSR2*	0	0	0	
Epigenetic modifiers	*ASXL1*	31 (24.8)	16 (23.2)	6 (31.6)	0.5509
	*EZH2*	2 (1.6)	0	0	
Transcription factors	*RUNX1*	11 (8.8)	6 (8.7)	0	0.3332
	*NPM1*	27 (21.6)	21 (14.5)	0	**0.0047**
	*CEBPA*	18 (14.4)	11 (15.9)	2 (10.5)	0.7256
	biallelic *CEBPA*	10 (8)	6 (8.7)	4 (22.2)	0.0882
	*ETV6*	5 (4)	3 (4.3)	0	
	*GATA2*	0	0	0	
Activited signaling	*FLT3*	29 (23.2)	16 (23.2)	0	**0.0184**
	*FLT3-ITD*	20 (16)	13 (18.8)	0	0.0624
	*FLT3-TKD*	11 (8.8)	3 (4.3)	0	
	*NRAS*	22 (17.6)	16 (23.2)	1 (5.3)	0.1054
	*KRAS*	0	0	0	
	*CBL*	1 (0.8)	0	1 (5.3)	
	*JAK2*	0	0	0	
	*KIT*	7 (5.6)	1 (1.4)	1 (5.3)	
Tumor suppressors	*CSF3R*	1 (0.8)	0	0	
	*TP53*	15 (12)	2 (2.9)	11 (57.9)	**<0.0001**
	*PHF6*	3 (2.4)	0	0	

### Clearance of mutations after induction treatment

**Figure 2:**
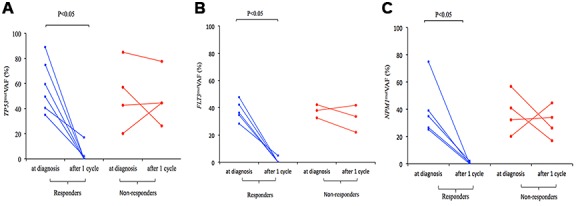
Change in *TP53*^mut^ (**A**), *FLT3*^mut^ (**B**), and *NPM1*^mut^ (**C**) VAF in responders and non-responders to D-CAG with paired samples at diagnosis and after 1 cycle.

We compared the frequency of mutations in the BM samples of 27 patients at diagnosis and after one cycle of induction treatment, and observed that clearance of leukemia-specific mutations correlated with morphological response and relapse. We observed significant reduction in variant allele frequency (VAF) of *TP53, NPM1 and FLT3* mutations among responders after one cycle of D-CAG treatment, but no significant change was observed among the non-responders (Figure 2). Persistence of these gene mutations correlated with worse outcomes. Driver mutations persisted after chemotherapy and increased in size at the time of relapse. New mutations were also found at relapse, thereby suggesting that disease progression was coupled with step-wise genetic evolution.

### Association between cytogenetics, gene mutations and clinical outcomes

After a median follow-up of 12 months (range: 2-82 months), 86 (68.8%) and 94 patients (75.2%) treated with D-CAG achieved CR after one cycle and two cycles of induction, respectively. Patients in the low- or intermediate-risk groups showed a higher CR rate than patients in the high-risk group, but the differences were not statistically significant (91.7% vs. 78.4% vs. 69.4%, P= 0.2053; Table 3).

**Table 3:** Response and clinical outcome.

**Clinical features**	**CR n (%)**	**Median OS (months)**	**Median DFS (months)**	**1-year OS (%)**	**2-year OS (%)**
All patients (n=125)	94 (75.2)	16	12	59.8	36.5
Age (years)	*P*=0.5473	*P*=**0.0070**	*P*=0.1220		
60-66 (n=65)	51 (78.5)	19	15	70.6	45.1
67-74 (n=39)	29 (74.4)	14	11	53.2	36.6
75-86 (n=21)	14 (66.7)	9	7	40.6	20.3
Patients aged 60-66 years (n=65)	*P*=0.3612	*P*=**0.0134**	*P*=0.4344		
Low- and intermediate-risk (n=37)	31 (83.8)	28	15	84.0	53.7
High-risk (n=28)	20 (71.4)	13	14	52.8	35.2
Patients aged 67-74 years (n=39)	*P*=1.0000	*P*=0.0681	*P*=**0.0014**		
Low- and intermediate-risk (n=17)	13 (76.5)	32	19	69.0	40.0
High-risk (n=22)	16 (72.7)	12	6	52.6	22.8
Patients aged 75-86 years (n=21)	*P*=0.6557	*P*=0.2110	*P*=0.1048		
Low- and intermediate-risk (n=8)	6 (75.0)	18	13	19.4	N/A
High-risk (n=13)	8 (61.5)	9	6	10.3	N/A
Numbers of mutations	*P*=0.0035	*P*=0.1570	*P*=0.6556		
0-1 (n=44)	26 (59.1)	12	18	47.0	26.9
2 (n=35)	32 (91.4)	18	11	69.6	39.3
≥3 (n=46)	36 (78.3)	17	14	64.2	34.7
*DNMT3A*	*P*=0.1208	*P*=0.6243	*P*=0.7438		
mut (n=24)	15 (62.5)	13	13	53.0	26.5
wt (n=101)	79 (78.2)	16	12	61.4	39.1
*TET2*	*P*=1632	*P*=0.6365	*P*=0.6875		
mut (n=33)	28 (84.8)	17	13	65.6	38.6
wt (n=92)	66 (71.7)	15	11	57.7	37.3
*IDH1*	*P*=0.7028	*P*=0.8188	*P*=0.2503		
mut (n=10)	7 (70)	Undefined	Undefined	51.4	N/A
wt (n=115)	87 (75.7)	16	12	60.5	37.6
*IDH2*	*P*=0.7603	*P*=0.1423	*P*=0.8574		
mut (n=15)	12 (80)	20	13	86.7	40.5
wt (n=110)	82 (74.5)	15	11	55.7	37.3
*ASXL1*	*P*=1.0000	*P*=0.4425	*P*=0.0801		
mut (n=31)	24 (77.4)	16	8	56.5	31.4
wt (n=94)	70 (74.5)	16	14	60.6	39.7
*RUNX1*	*P*=1.0000	*P*=0.7141	*P*=0.4392		
mut (n=11)	8 (72.7)	18	18	71.6	42.9
wt (n=114)	86 (75.4)	16	12	58.7	37.5
*NPM1*	*P*=0.4602	*P*=0.3479	*P*=0.1092		
mut (n=27)	22 (81.5)	19	18	65.6	41.4
wt (n=98)	72 (73.5)	15	10	57.8	36.8
*CEBPA*	*P*=0.3451	*P*=0.2662	*P*=0.0994		
biallelic mut (n=10)	9 (90)	15.5	9.5	60.0	20.0
monoallelic mut (n=8)	7 (87.5)	19	18	75.0	46.9
wt (n=107)	78 (72.9)	16	12	58.8	39.9
*FLT3*	*P*=0.8065	*P*=0.2247	*P*=0.8653		
mut (n=29)	21 (72.4)	12	18	46.8	32.8
wt (n=96)	73 (76.0)	17	12	63.3	37.2
*NRAS*	*P*=1.0000	*P*=0.7962	*P*=0.7377		
mut (n=22)	17 (77.3)	28	19	58.7	52.8
wt (n=103)	77 (74.8)	15	11	60.0	34.9
*KIT*	*P*=1.0000	*P*=0.4152	*P*=0.1337		
mut (n=7)	5 (71.4)	14	7.5	60.0	N/A
wt (n=118)	89 (75.4)	16	12	59.7	39.2
*TP53*	*P*=1.0000	*P*=0.0657	*P*=0.0649		
mut (n=15)	11 (73.3)	10	7	46.7	15.6
wt (n=110)	83 (75.5)	18	14	66.3	39.7
Cytogenetics					
	*P*=0.5641	*P*=**0.0041**	*P*=**0.0001**		
complex karotypes (n=19)	13 (68.4)	9	5	35.5	11.8
others (n=106)	81 (76.4)	19	15	64.6	41.5
	*P*=0.7713	*P*<**0.0001**	*P*<**0.0001**		
-5/5q-, -7/7q- (n=18)	13 (72.2)	8.5	6	27.8	5.6
others (n=107)	81 (75.7)	19	15	65.8	44.5
	*P*=0.2911	*P*=0.3143	*P*=**0.0004**		
monosomal (n=12)	11 (91.7)	13	5	57.1	19.0
others (n=113)	83 (73.5)	17	14	60.1	40.3
risk status	*P*=0.3727	*P*=**0.0022**	*P*=**0.0041**		
Low-risk (n=12)	11 (91.7)	Undefined	15	88.9	71.1
Intermediate-risk (n=50)	39 (78.0)	20	15	74.0	47.9
High-risk (n=63)	44 (69.8)	11	8	44.1	25.8

Abbreviations: CR: complete remission; OS: overall survival; DFS: disease-free survival; mut: mutated status; wt: wild type.

**Figure 3:**
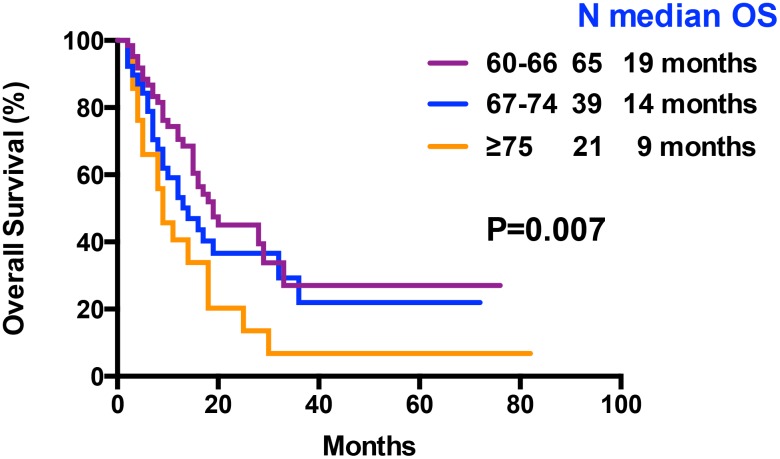
**Kaplan–Meier curves associated with overall survival within age arms (60-66 vs 67-74 vs ≥ 75 years).**

**Figure 4:**
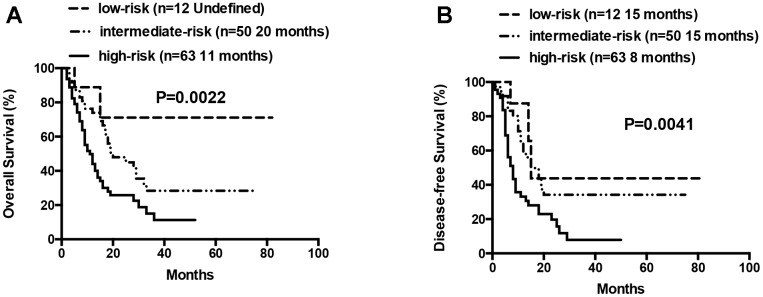
**Overall survival and disease free survival according to risk groups (low-risk vs intermediate-risk vs high-risk).** (**A**) Overall survival in low-, intermediate- and high-risk patients. (**B**) Disease free survival in low-, intermediate- and high-risk patients.

**Figure 5:**
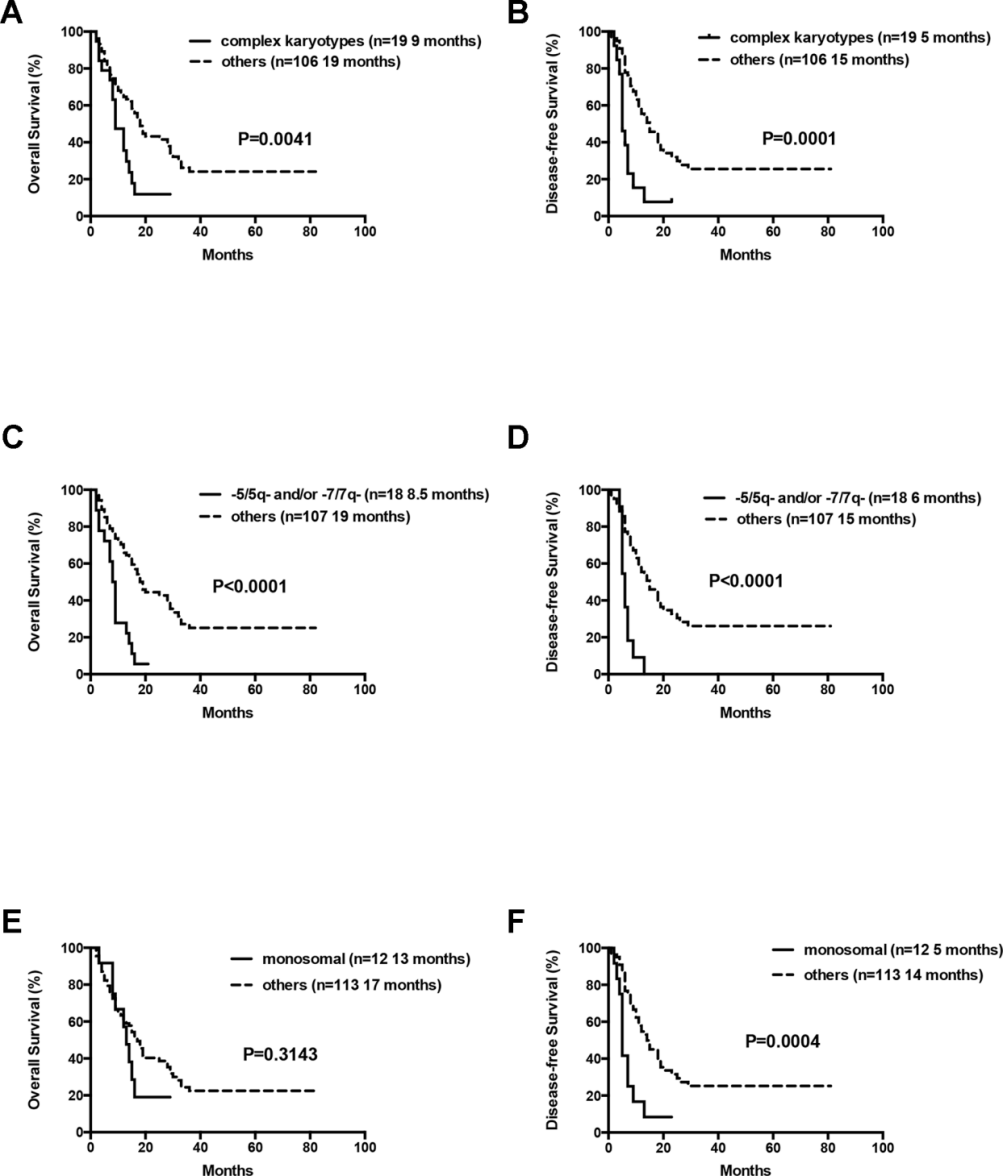
**Overall survival and disease free survival according to cytogenetics.** (**A**) Overall survival in AML patients with complex cytogenetics compared to others. (**B**) Disease free survival in AML patients with complex cytogenetics compared to others. (**C**) Overall survival in AML patients with abnormalities in -5/5q- and/or -7/7q- chromosomal deletions compared to others. (**D**) Disease free survival in AML patients with abnormalities in -5/5q- and/or -7/7q- chromosomal deletions compared to others. (**E**) Overall survival in AML patients with monosomal karyotype compared to others. (**F**) Disease free survival in AML patients with monosomal karyotype compared to others.

At the final analysis on July 30, 2019, the median OS and DFS of all patients were 16 and 12 months, respectively. The median OS was 19, 14 and 9 months for patients aged 60–66 years, 67–74 years, and ≥75 years, respectively (P=0.007, Figure 3). The one-year and 2-year OS rates were 59.8% and 36.5%, respectively. The one-year and 2-year DFS rates were 49.3% and 28.2%, respectively. Patients aged 60–66 years showed significantly longer OS than patients ≥75 years (median OS: 19 months vs. 9 month, P= 0.0042, Table 3, Figure 3). However, DFS was statistically similar for both groups (median DFS: 15 months vs. 7 months, P= 0.0526, Table 3). The OS and DFS rates were significantly longer in patients belonging to the low- and intermediate-risk groups than in patients belonging to the high-risk group (median OS: Undefined vs. 20 months vs. 11 months, P= 0.0022; median DFS: 15 months vs. 15 months vs. 8 months, P= 0.0041, respectively; Table 3, Figure 4). Patients with complex karyotypes showed significantly shorter median OS and DFS compared to patients with non-complex karyotypes (n=19; median OS: 9 months vs. 19 months, P= 0.0041; median DFS: 5 months vs. 15 months, P= 0.0001, respectively; Table 3, Figure 5). Moreover, patients with abnormalities in chromosomes 5 and/or 7 (-5/5q- and/or -7/7q-) showed significantly shorter median OS and DFS compared to patients without these abnormalities (-5/5q- and/or -7/7q-, n=18; median OS: 8.5 months vs. 19 months, P< 0.0001; median DFS: 6 months vs. 15 months, P< 0.0001, respectively; Table 3, Figure 5). Patients with monosomal karyotypes (n=12) showed similar median OS, but significantly shorter median DFS compared to other patients (median OS: 13 months vs. 17 months, P= 0.3143; median DFS: 5 months vs. 14 months, P= 0.0004; Table 3, Figure 5).

**Figure 6:**
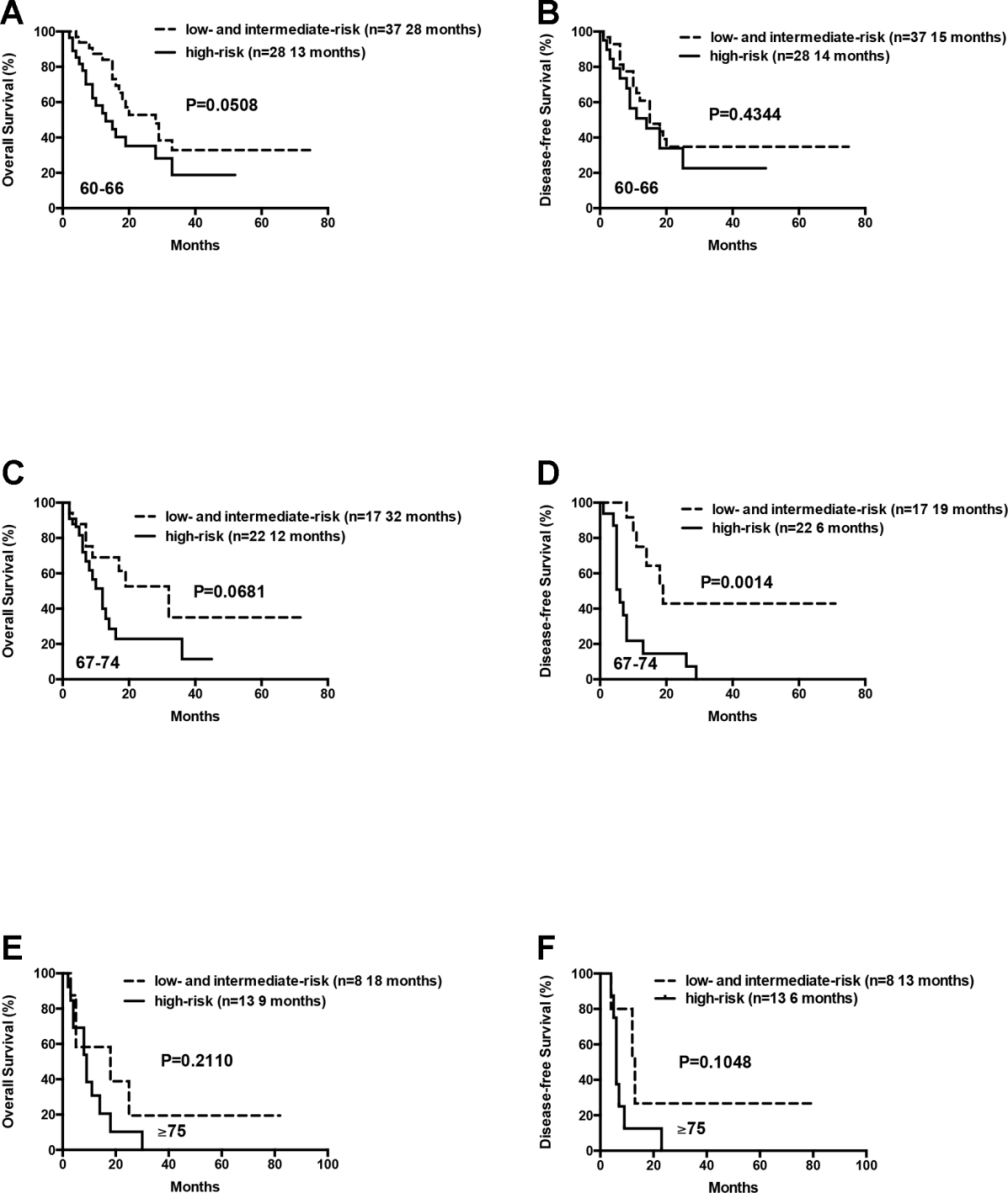
**Overall survival and disease free survival of the AML patients according to risk groups (favorable and intermediate vs poor) within age arms (60-66 vs 67-74 vs ≥ 75 years).** (**A**) Overall survival of the AML patients aged 60-66 years according to risk groups. (**B**) Disease free survival of the AML patients aged 60-66 years according to risk groups. (**C**) Overall survival of the AML patients aged 67-74 years according to risk groups. (**D**) Disease free survival of the AML patients aged 67-74 years according to risk groups. (**E**) Overall survival of the AML patients aged ≥75 years according to risk groups. (**F**) Disease free survival of the AML patients aged ≥75 years according to risk groups.

Patients in the 60-66 and 67-74 age groups showed better OS and DFS for the low- and intermediate-risk group patients compared to the high-risk group patients. But, these differences were not statistically significant except that median DFS for the low- and intermediate-risk groups was significantly longer than the high-risk group for patients aged 67-74 years (median DFS: 19 months vs. 6 months, P= 0.0014; Figure 6).

We also analyzed 21 patients who were ≥75 years, including 8 low- or intermediate-risk patients and 13 high-risk patients. The median OS and DFS were relatively longer in the low- and intermediate-risk group compared to the high-risk group, but the differences were not statistically significant (median OS: 18 months vs. 9 months, P= 0.2110; median DFS: 13 months vs. 6 months, P= 0.1048; Figure 6). This suggests that D-CAG is feasible for the treatment of AML patients above 75 years, especially those harboring high-risk karyotypes and genetic mutations.

**Figure 7:**
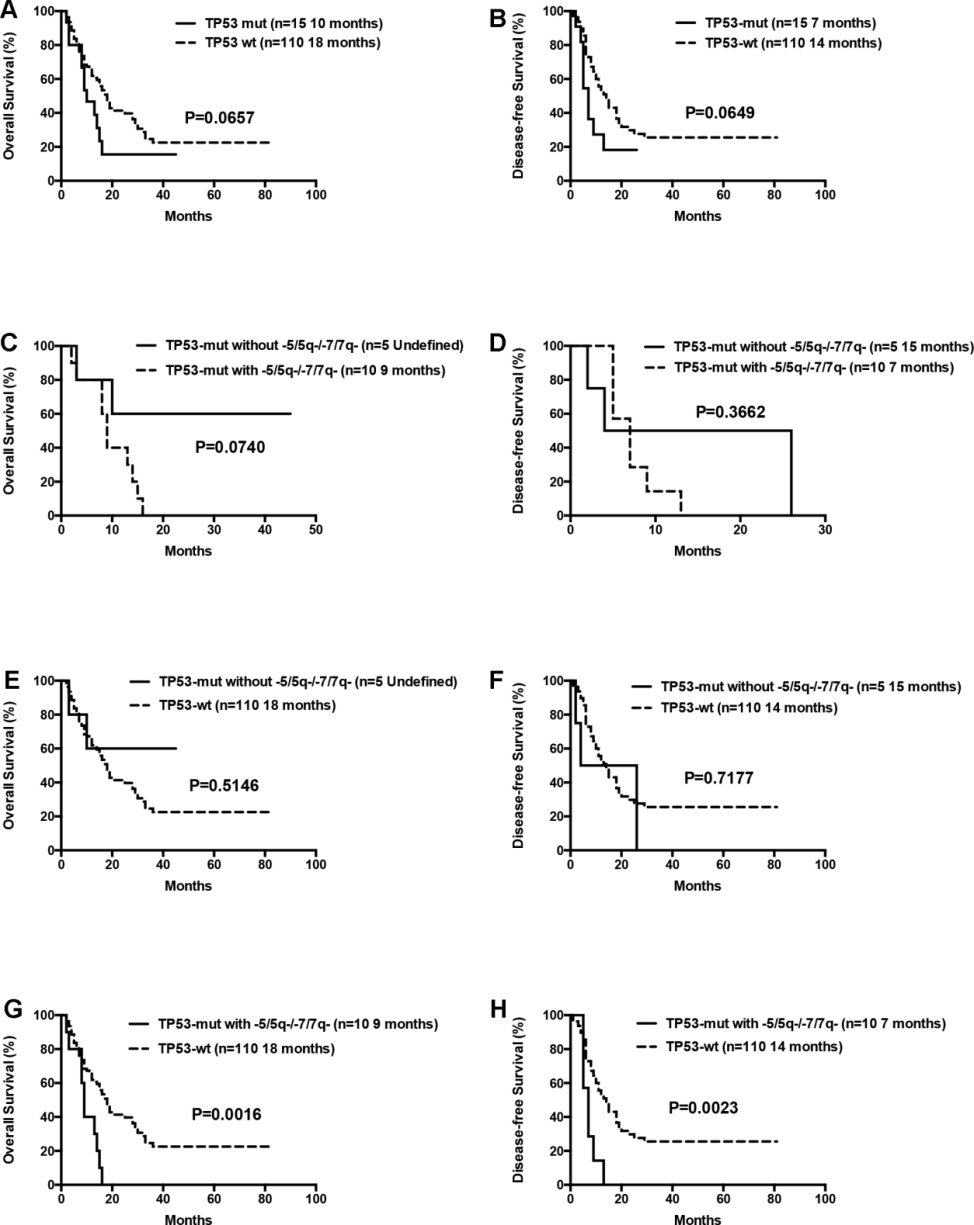
**Overall survival and disease free survival according to *TP53* mutations.** (**A**) Overall survival in *TP53* mutated compared to *TP53* wild-type patients. (**B**) Disease free survival in *TP53* mutated compared to *TP53* wild-type patients. (**C**) Overall survival in isolated *TP53* mutated patients compared to those with *TP53* mutations and concomitant -5/5q- and/or -7/7q- chromosomal deletions. (**D**) Disease free survival in isolated *TP53* mutated patients compared to those with *TP53* mutations and concomitant -5/5q- and/or -7/7q- chromosomal deletions. (**E**) Overall survival in isolated *TP53* mutated compared to *TP53* wild-type patients. (**F**) Disease free survival in isolated *TP53* mutated compared to *TP53* wild-type patients. (**G**) Overall survival in patients with *TP53* mutations and concomitant -5/5q- and/or -7/7q- chromosomal deletions compared to *TP53* wild-type patients. (**H**) Disease free survival in patients with *TP53* mutations and concomitant -5/5q- and/or -7/7q- chromosomal deletions compared to *TP53* wild-type patients.

**Figure 8:**
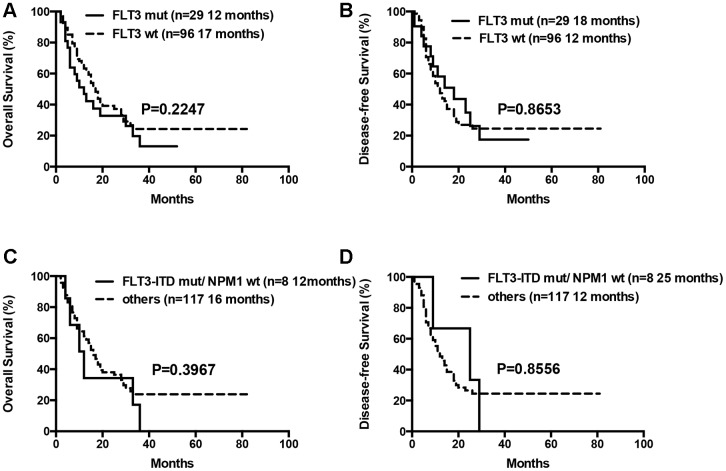
**Overall survival and disease free survival according to *FLT3* mutations.** (**A**) Overall survival in *FLT3* mutated compared to *FLT3* wild-type patients. (**B**) Disease free survival in *FLT3* mutated compared to *FLT3* wild-type patients. (**C**) Overall survival in patients with *FLT3-ITD* mutations in the absence of *NPM1* mutations compared to others. (**D**) Disease free survival in patients with *FLT3-ITD* mutations in the absence of *NPM1* mutations compared to others.

The median OS for patients with 0 or 1, 2 or ≥3 gene mutations was not statistically significant (12 months vs. 18 months vs. 17 months, P= 0.1570). Moreover, OS and DFS for patients with or without mutations in genes such as *ASXL1* and *RUNX1* were similar (Table 3). Patients with wild-type *TP53* (n=110) showed relatively longer OS and DFS compared to patients harboring *TP53* mutations (n=15), but the differences were not statistically significant (median OS: 18 months vs. 10 months, P= 0.0657; median DFS: 14 months vs. 7 months, P= 0.0649; Table 3, Figure 7). Patients with *TP53* mutations (VAF <20%) showed relatively longer survival than patients with *TP53* mutations (VAF ≥20%), but, the data was not statistically significant after excluding two patients with low VAF (<20%) *TP53* mutations (median OS: 18 months vs. 9 months, P= 0.1047; median DFS: 14 months vs. 7 months, P= 0.0511). Ten out of 15 patients with *TP53* mutations were associated with -5/5q- and/or -7/7q- chromosomal deletions, whereas, the remaining 5 patients had isolated *TP53* mutations. The patients with isolated *TP53* mutations showed relatively longer median OS and DFS compared to patients with *TP53* mutations and concomitant -5/5q- and/or -7/7q- chromosomal deletions (median OS: undefined vs. 9 months, P= 0.0740; median DFS: 15 months vs. 7 months, P= 0.3662; Table 3, Figure 7). The median OS and DFS was comparatively similar for patients with wild-type and isolated *TP53* mutations (median OS: 18 months vs. undefined, P= 0.5146; median DFS: 14 months vs. 15 months, P= 0.7177; Table 3, Figure 7), but, significantly lower in patients with *TP53* mutations and concomitant -5/5q- and/or -7/7q- chromosomal deletions (median OS: 9 months vs. 18 months, P= 0.0016; median DFS: 7 months vs. 14 months, P= 0.0023, respectively; Table 3, Figure 7). The median OS and DFS was statistically similar for AML patients with or without *FLT3* mutations (median OS: 12 months vs. 17 months, P= 0.2247; median DFS: 18 months vs. 12 months, P= 0.8653, respectively; Table 3, Figure 8). Patients with *FLT3-ITD* mutations in the absence of *NPM1* mutations (n=8) and the other patients (n=117) showed statistically similar median OS and DFS (median OS: 12 months vs. 16 months, P= 0.3967; median DFS: 25 months vs. 12 months, P= 0.8556, Figure 8).

Univariable analyses showed that age (≥75 years), complex karyotypes, -5/5q- and/or -7/7q- chromosomal deletions, and high-risk status were independent prognostic factors associated with shorter OS (Table 4). Factors such as monosomal karyotypes, total number of gene mutations, mutated *TP53* and *FLT3*, and *FLT3-ITD* mutations in the absence of *NPM1* mutations did not show prognostic significance in the univariable analysis. Multivariate analysis showed that age (over 75 years), high-risk status, and -5/5q- and/or -7/7q- chromosomal deletions were significant variables that predicted poor prognosis or decreased OS (Table 4).

**Table 4:** Univariate and multivariate analysis.

**Variable**	**Univariate analysis**		**Multivariate analysis**	
	**HR (95% CI)**	**P**	**HR (95% CI)**	**P**
OS				
age (≥75 yrs vs <75 yrs)	1.995 (1.159~3.435)	**0.013**	1.901 (1.099~3.288)	**0.022**
complex karotypes	2.209 (1.254~3.893)	**0.006**	0.689 (0.252~1.887)	0.469
-5/5q- and/or -7/7q-	3.268 (1.855~5.757)	**<0.001**	3.206 (1.157~8.885)	**0.025**
monosomal karyotypes	1.421 (0.704~2.871)	0.327	N/A	N/A
risk status	1.967 (1.310~2.952)	**0.001**	1.620 (1.058~2.482)	**0.027**
numbers of mutations (0-1)	N/A	0.174	N/A	N/A
numbers of mutations (2)	1.461 (0.863~2.473)	0.158	N/A	N/A
numbers of mutations (≥3)	0.889 (0.496~1.593)	0.693	N/A	N/A
Mutations				
*DNMT3A*	1.148 (0.652~2.021)	0.631	N/A	N/A
*TET2*	1.125 (0.717~1.764)	0.609	N/A	N/A
*IDH1*	0.891 (0.325~2.442)	0.822	N/A	N/A
*IDH2*	0.587 (0.282~1.224)	0.155	N/A	N/A
*ASXL1*	1.217 (0.729~2.032)	0.453	N/A	N/A
*RUNX1*	0.858 (0.373~1.977)	0.72	N/A	N/A
*NPM1*	0.767 (0.435~1.352)	0.359	N/A	N/A
*CEBPA*	0.959 (0.527~1.745)	0.892	N/A	N/A
*FLT3*	1.370 (0.814~2.306)	0.236	N/A	N/A
*FLT3-ITD* mut/NPM1 wt	1.424 (0.616~3.292)	0.408	N/A	N/A
*NRAS*	0.926 (0.509~1.684)	0.8	N/A	N/A
*KIT*	1.507 (0.547~4.151)	0.428	N/A	N/A
*TP53*	1.759 (0.944~3.277)	0.075	N/A	N/A

Abbreviations: OS: overall survival; HR: hazard ratio.

### Adverse events

The most frequently observed adverse events during D-CAG therapy were myelosuppression and infections. All the AML patients treated with D-CAG experienced thrombocytopenia and neutropenia. Febrile neutropenia occurred in 82.4% of the patients, but sepsis was reported only in 6 cases. Non-hematological toxicities were usually mild to moderate (Table 5). For patients achieving CR, the median times for showing stable neutrophil (0.5×10^9^ neutrophils/L) and platelet (20×10^9^/L) counts were 14 days and 16 days, respectively.

**Table 5:** Toxicities.

	**Grade 1-2 n (%)**	**Grade 3-4 n (%)**
Hematological Toxicities		
Thrombocytopenia	7 (5.6)	118 (94.4)
Neutropenia	18 (14.4)	107 (85.6)
Febrile neutropenia	74 (59.2)	29 (23.2)
Non-hematological Toxicities		
Hepatobiliary disorders	27 (21.6)	3 (2.4)
Nausra, Vomiting	22 (17.6)	0
Mucositis	26 (20.8)	0
Skin disorders	3 (2.4)	0
Cardiac disorders	7 (5.6)	0
Sepsis	6 (4.8)	0

## DISCUSSION

The poor prognosis in elderly AML patients is attributed to several factors, such as, high frequency of adverse cytogenetics, higher incidence of early deaths during chemotherapy, and higher rates of drug resistance and cancer relapse [12]. The optimal regimen to treat elderly AML patients still remains controversial. Elderly patients receiving standard or intensive chemotherapy experience more frequent treatment-related mortality (TRM) than younger patients because of myelosuppression, infections, and hemorrhage [13–15]. The risk of early death in elderly AML patients undergoing standard-dose induction chemotherapy is about 10-20% [16]. Eisfeld A. K. et al reported a CR rate of 81%, 50% and 32% in response to standard chemotherapy in favorable-, intermediate- and high-risk elderly AML patients, respectively; 3-year OS and DFS rates for patients belonging to the favorable-risk group were 45% and 46%, respectively; however, the 3-year OS and DFS rates for patients with adverse genetic features were only 4% and 2%, respectively [17]. Numerous *FLT3* tyrosine kinase inhibitors (TKIs) have been developed over the last several years to treat AML patients with *FLT3* mutations. Older patients treated with *FLT3* TKIs in combination with hypomethylating agents (HMA) show an overall response rate (ORR) between 26% and 67% [18–20]. Venetoclax, an oral BCL-2 inhibitor, has been approved for the treatment of older AML patients ineligible for intensive chemotherapy by the United States Food and Drug Administration (FDA) in combination with low-dose cytarabine (LDAC) or hypomethylating agents (HMA) such as azacitidine and decitabine. The CR plus CR with incomplete hematological recovery rates for older patients treated with venetoclax plus LDAC or HMA are 54% and 67%, respectively, and the median OS are 10.4 months and 17.5 months, respectively, which are significantly higher compared with clinical outcomes when treated with LDAC or HMA alone [21, 22]. The patients analyzed in this study did not receive any treatment with *FLT3* TKI inhibitors and/or venetoclax because these medications have not yet been approved in China for AML treatment.

The standard treatment regimen for elderly AML patients includes hypomethylating agents, such as decitabine or azacitidine in combination with chemotherapeutic agents [23, 24]. In our previous clinical study, we demonstrated improved clinical outcomes in elderly AML patients treated with D-CAG regimen; CR rates were comparable for patients with or without adverse karyotypes; OS was comparatively similar for patients aged ≥70 years old and 60–69 years when treated with D-CAG induction therapy [8]. *In vitro* experiments show that decitabine and cytarabine synergistically induce apoptosis of human leukemia cell lines [25]. Genome-wide studies show that aclarubicin uniquely alters the epigenetic gene expression in mouse and human leukemia cells via histone eviction [26]. Aclarubicin accumulates in the mitochondria and inhibits respiratory function, thereby causing mitochondrial dysfunction, which promotes cytotoxicity [27]. Thus, D-CAG is a regimen that synergistically employs multiple mechanisms to block the growth and survival of AML cells.

NGS assays have been recently integrated into the routine laboratory work-up of AML cases at our center because of its ultra-high throughput, scalability, and speed. However, our previous studies lacked in-depth analysis of the genetic mutations. We investigated the significance of mutations on clinical outcomes in elderly AML patients that were treated with the D-CAG regimen. The risk status was defined according to the updated NCCN guidelines for AML by performing NGS assay of 23 selected genes and cytogenetic profiling of BM samples of elderly patients with AML before treatment. The elderly AML patients, unlike younger patients, are associated with a higher percentage of adverse karyotypes and mutations. In our study, nearly 15.2% elderly patients showed complex karyotypes and approximately 50% of the patients was classified as high-risk.

In our study, patients with complex karyotypes and high-risk status were associated with shorter OS. Univariate analysis shows that complex karyotypes and high-risk status are independent prognostic factors for OS. Moreover, the median OS and DFS of the high-risk patients above 75 years old are relatively shorter than those with low- and intermediate-risk status, but the differences are not statistically significant. This indicates that the D-CAG regimen might improve the prognosis and OS of elderly patients over 75 years in the high-risk group.

We observed a higher proportion of mutations in the *TET2*, *DNMT3A* and *IDH2* genes in the elderly patients analyzed in our study. Mutations in the genes encoding epigenetic modifiers, such as *TET2*, *DNMT3A* and *IDH2*, are more common in older patients, and are usually acquired early in the evolution of the disease, and often present in the founding clone. In this study, the median OS and DFS was comparable for patients with mutated or wild type *TET2*, *DNMT3A* and *IDH2*.

Approximately 5-8% of all patients with AML harbor *TP53* mutations. In this study, 12% of the older patients showed *TP53* mutations, suggesting that these mutations are higher in the older AML patients. Hematological malignancies carrying *TP53* mutations, abnormalities in chromosomes 5, 7, or 17p, and complex karyotypes are associated with poor prognosis, regardless of the treatment choice. The median OS was 7.2 and 2.4 months, respectively, for elderly AML patients with *TP53* mutations treated with azacitidine or conventional care [23]. The median OS in elderly patients with AML and *TP53* mutations receiving standard cytotoxic chemotherapy is 4 to 6 months [28–30]. In our study, 15 patients with *TP53* mutations had a median OS of 10 months, which was relatively shorter than the median OS of 18 months for patients with wild type *TP53*, but the difference was not statistically significant. Lower VAF is associated with better survival in elderly AML patients with *TP53* mutations [31, 32]. We performed survival analysis after excluding the two patients harboring *TP53* mutations with VAF <20%, and did not observe statistical differences between patients with mutated and wild-type *TP53*. In our study, ten patients with *TP53* mutations also harbored -5/5q- and/or -7/7q- chromosomal deletions, whereas, the remaining 5 patients had isolated *TP53* mutations. The median OS and DFS was relatively longer for patients with isolated *TP53* mutations compared to patients with *TP53* mutations and concomitant -5/5q- and/or -7/7q- chromosomal deletions, thereby indicating worse prognosis for the latter group. Complex cytogenetics, monosomal karyotypes, -5/5q- and/or -7/7q- chromosomal deletions, and *TP53* mutations frequently overlap with each other. Since only patients with -5/5q- and/or -7/7q- chromosomal deletions were associated with poorer survival in the multivariate analysis, we speculate that D-CAG tends to improve the prognosis of *TP53* mutated patients.

Approximately 20% of AML patients harbor *FLT3* mutations that are more common in younger patients with normal karyotype, and is associated with poor prognosis [33]. Dohner et al*.* reported that OS was significantly lower in the azacitidine-treated older AML patients with *FLT3* mutations compared to those undergoing conventional care [23]. In our previous study, OS was significantly lower in eleven patients with *FLT3-ITD* mutations compared to other patients [8]. In this study, we detected *FLT3* mutations in 23.2% of the patients using NGS. The median OS was similar for patients with or without *FLT3* mutations. We further analyzed survival of eight patients with *FLT3-ITD* and without *NPM1* mutations, and found no significant differences in OS and DFS between patients with the mutated gene and the others. Patients with persistent mutations after chemotherapy showed significantly lower event-free survival and OS than those that cleared all mutations [34]. We confirmed clearance of *TP53* and *FLT3* mutations in some responders, thereby confirming that D-CAG eliminates mutant clones of *TP53* and *FLT3*.

The limitations of this study include small sample size, and limited analysis of pre-treatment, post-treatment, and relapsed samples for all cases. While our study indicates that D-CAG treatment regimen improves overall prognosis of elderly patients with AML, the findings need to be validated by prospective studies with larger cohort of patients and a longer follow-up.

In conclusion, our study provides evidence for the clinical efficacy of the D-CAG treatment regimen in older AML patients and shows that D-CAG tends to improve the prognosis of a subgroup of elderly patients with high-risk AML.

## MATERIALS AND METHODS

### Study populations

We enrolled 125 patients between the ages 60-86 years that were diagnosed with AML (except acute promyelocytic leukemia) in our hospital between September 2011 and October 2018 according to the WHO classification. The Eastern Cooperative Oncology Group Performance Status (ECOG PS) score of all patients was 0-3. The exclusion criteria included poor hepatic or renal function, defined as total serum bilirubin, aminotransferase, or creatinine concentrations more than two times the upper limit of normal range; poor cardiac function greater than Class II according to the New York Heart Association Functional Classification; presence of another malignancy without remission. The included patients had not received any previous chemotherapy other than hydroxyurea. The study procedures and informed consent forms were approved by the ethics committee of the First Affiliated Hospital of Nanjing Medical University, Jiangsu Province Hospital (number 2011-SR-085). We obtained informed consent from all patients included in this study or their legal guardians.

This study was registered on the Chinese Clinical Trial Registry (ChiCTR No. 11001700). The target accrual number for this study was 100 and the duration was from September 2011 to September 2016. We enrolled 89 patients in this observational study (ChiCTR number 1001700), including 81 patients that participated in our previous study [8]. The remaining 36 patients came from an extension cohort, including 16 patients aged above 75 years. They were not enrolled in the clinical trial and were treated with D-CAG according to the 2017 Chinese guidelines for diagnosis and treatment of adult acute myeloid leukemia [35]. These guidelines required AML patients above 60 years to receive decitabine combined with low-dose chemotherapeutic regimen, such as CAG.

### D-CAG treatment regimen

The patients were treated with the D-CAG regimen as previously reported [8]. Briefly, patients were intravenously injected with 15 mg/m^2^ decitabine over 4 h for five consecutive days (days 1-5), 10 mg/m^2^ cytarabine every 12h for seven days (days 3-9), 10 mg/m^2^/day aclarubicin for four days (days 3-6) from October 2010 to April 2016 or 8 mg/m^2^/day aclarubicin for four days (days 3-6) from May 2016 to May 2018, and 300 μg/day G-CSF for priming until the white blood cell (WBC) counts exceeded 20×10^9^/L. Up to two cycles of induction chemotherapy were allowed if CR was not achieved. Patients who did not achieve CR after two cycles of D-CAG were offered alternative therapies or supportive therapy. The post-remission therapy included 4–6 cycles of D-CAG for the patients achieving CR. Upon final analysis on July 30, 2019, D-CAG as post-remission therapy was administrated in 89 patients for one cycle, 74 patients for two cycles, 40 patients for three cycles, 33 patients for four cycles, 12 patients for 5 cycles and 5 patients for 6 cycles, respectively.

### Cytogenetic analyses

Bone marrow (BM) cells were directly harvested from unstimulated cultures. Metaphase cells were banded using an improved heat treatment and Giemsa R-banding method. The karyotyping was based on conventional cytogenetic examination of ≥20 metaphases.

### Next-generation sequencing

A total of 23 genes, including *DNMT3A*, *TET2*, *IDH1*, *IDH2*, *SRSF2*, *U2AF1*, *ZRSR2*, *ASXL1*, *EZH2*, *RUNX1*, *NPM1*, *CEBPA*, *ETV6*, *GATA2*, *FLT3*, *NRAS*, *KRAS*, *CBL*, *JAK2*, *KIT*, *CSF3R*, *TP53* and *PHF6*, were included in the targeted gene sequencing (TGS) panel. Genomic DNA (gDNA) was extracted from the BM aspirates of each patient using an Autopure extractor (Qiagen, Hilden, Germany). Then, 10 ng genomic DNA was PCR amplified using the Ion AmpliSeq Library kit 2.0 (Ion Torrent, Thermo Fisher Scientific, USA). The 206 amplicons included in this panel are summarized in Supplementary Table 1. Amplicon libraries were constructed using the KAPA Hyper Prep kit for Illumina Platforms (Kapa Biosystems, Wilmington, MA, USA) [36], and sequenced on the Illumina Miseq platform. The sequencing reads were aligned to the human reference genome (genome build hg19) using the Burrows-Wheeler Aligner (BWA) [37]. Variant calling was performed using the Genome Analysis Toolkit (GATK) [38]. The Integrative Genomics Viewer (IGV) version 2.3.32 was used to visualize the sequencing reads and assess the variants [39]. We excluded from further analysis the synonymous and non-synonymous variants that occurred with a frequency >0.1% in the 1000 Genomes Project database (http://www.ncbi.nlm.nih.gov/variation/tools/1000genomes/) or in the normal Eastern Asian population from the Exome Aggregation Consortium (ExAC; http://exac.broadinstitute.org/).

### Definition of outcomes

Treatment responses were assessed according to the NCCN clinical practice guidelines of AML (version 2.2019). The complete remission (CR) was defined by transfusion independence, <5% blasts in the BM aspirate with spicules, absence of blasts with Auer rods, absence of residual extramedullary disease, as well as absolute neutrophil counts >1.0×10^9^/L, and platelet counts ≥100×10^9^/L in peripheral blood. Partial remission (PR) was defined by a 50% decrease in the percentage of blasts to 5-25% in the BM aspirate, and the normalization of blood counts as described above. No remission (NR) was indicated when the CR or PR criteria were not met as described above. The objective response rate (ORR) included rates of CR and PR. The overall survival (OS) was measured from the time of diagnosis to death or censorship at the last follow-up. The disease-free survival (DFS) was defined as the duration from CR until relapse or death or censorship at the last follow-up. The time to stable neutrophil recovery was measured from the end of protocol induction therapy until the first day when the absolute neutrophil counts recovered to ≥500/μl for two consecutive measurements on different days. The time to stable platelet recovery was measured from the end of protocol induction therapy until the first day that the platelet counts were ≥20,000/μl for at least seven consecutive days. Toxicities were defined and graded according to the National Cancer Institute (NCI) Common Toxicity Criteria [40].

### Statistical analysis

Data were analyzed using the Statistical Package for Social Sciences (SPSS version 20.0). Statistical significance was considered when the *p* value was <0.05. Differences in continuous variables were analyzed by t-tests and chi-squared tests. Fisher exact test was performed to compare the incidences. Kaplan–Meier analysis was performed to estimate the survival probabilities, and proportional hazards model was used for univariate and multivariate analysis.

## Supplementary Material

Supplementary Table 1
