# Supplementary material for: Decitabine in combination with low-dose cytarabine, aclarubicin and G-CSF tends to improve prognosis in elderly patients with high-risk AML
Source: Aging (Albany NY). 2020 Apr 1;12(7):5792–811. doi: 10.18632/aging.102973 (PMC7185116; doi:10.18632/aging.102973)
Supplement: Supplementary Table 1 [file aging-12-102973-s001..docx]

**Supplementary Table 1. Amplicons.**

| **Chromosome** | **Gene** | **Starting point on the chromosome** | **Ending point on the chromosome** |
| --- | --- | --- | --- |
| chr17 | *TP53* | 7576972 | 7577196 |
| chr17 | *TP53* | 7577398 | 7577615 |
| chr17 | *TP53* | 7578087 | 7578296 |
| chr17 | *TP53* | 7578209 | 7578421 |
| chr17 | *TP53* | 7578385 | 7578554 |
| chr12 | *KRAS* | 25378561 | 25378663 |
| chr12 | *KRAS* | 25380260 | 25380337 |
| chr12 | *KRAS* | 25398183 | 25398385 |
| chr2 | *DNMT3A* | 25457048 | 25457271 |
| chr2 | *DNMT3A* | 25457266 | 25457438 |
| chr2 | *DNMT3A* | 25458501 | 25458729 |
| chr2 | *DNMT3A* | 25461879 | 25462103 |
| chr2 | *DNMT3A* | 25463103 | 25463329 |
| chr2 | *DNMT3A* | 25463445 | 25463664 |
| chr2 | *DNMT3A* | 25466627 | 25466857 |
| chr2 | *DNMT3A* | 25466845 | 25467042 |
| chr2 | *DNMT3A* | 25467042 | 25467265 |
| chr2 | *DNMT3A* | 25467937 | 25468158 |
| chr2 | *DNMT3A* | 25468148 | 25468373 |
| chr2 | *DNMT3A* | 25468816 | 25469022 |
| chr2 | *DNMT3A* | 25470424 | 25470643 |
| chr2 | *DNMT3A* | 25505266 | 25505486 |
| chr2 | *DNMT3A* | 25505375 | 25505580 |
| chr13 | *FLT3* | 28592526 | 28592736 |
| chr13 | *FLT3* | 28607944 | 28608146 |
| chr13 | *FLT3* | 28608136 | 28608351 |
| chr20 | *ASXL1* | 31022172 | 31022395 |
| chr20 | *ASXL1* | 31022369 | 31022591 |
| chr20 | *ASXL1* | 31022586 | 31022704 |
| chr20 | *ASXL1* | 31022694 | 31022850 |
| chr20 | *ASXL1* | 31022840 | 31022970 |
| chr20 | *ASXL1* | 31022960 | 31023162 |
| chr20 | *ASXL1* | 31023152 | 31023379 |
| chr20 | *ASXL1* | 31023369 | 31023585 |
| chr20 | *ASXL1* | 31023575 | 31023763 |
| chr20 | *ASXL1* | 31023752 | 31023954 |
| chr20 | *ASXL1* | 31023943 | 31024159 |
| chr20 | *ASXL1* | 31024149 | 31024325 |
| chr20 | *ASXL1* | 31024315 | 31024531 |
| chr20 | *ASXL1* | 31024521 | 31024642 |
| chr20 | *ASXL1* | 31024632 | 31024847 |
| chr20 | *ASXL1* | 31024837 | 31024967 |
| chr20 | *ASXL1* | 31024957 | 31025108 |
| chr20 | *ASXL1* | 31025098 | 31025231 |
| chr11 | *WT1* | 32413419 | 32413642 |
| chr11 | *WT1* | 32417766 | 32417991 |
| chr19 | *CEBPA* | 33792139 | 33792354 |
| chr19 | *CEBPA* | 33792278 | 33792486 |
| chr19 | *CEBPA* | 33792478 | 33792688 |
| chr19 | *CEBPA* | 33792702 | 33792835 |
| chr19 | *CEBPA* | 33792824 | 33792994 |
| chr19 | *CEBPA* | 33793011 | 33793112 |
| chr19 | *CEBPA* | 33793102 | 33793322 |
| chr19 | *CEBPA* | 33793311 | 33793455 |
| chr21 | *RUNX1* | 36164535 | 36164731 |
| chr21 | *RUNX1* | 36164686 | 36164881 |
| chr21 | *RUNX1* | 36171591 | 36171810 |
| chr21 | *RUNX1* | 36206706 | 36206885 |
| chr21 | *RUNX1* | 36206885 | 36206960 |
| chr21 | *RUNX1* | 36231716 | 36231936 |
| chrX | *BCOR* | 39913101 | 39913326 |
| chrX | *BCOR* | 39931583 | 39931744 |
| chrX | *BCOR* | 39931734 | 39931962 |
| chrX | *BCOR* | 39931952 | 39932172 |
| chrX | *BCOR* | 39932162 | 39932388 |
| chrX | *BCOR* | 39932378 | 39932558 |
| chrX | *BCOR* | 39932548 | 39932705 |
| chrX | *BCOR* | 39932695 | 39932850 |
| chrX | *BCOR* | 39932844 | 39933029 |
| chrX | *BCOR* | 39933019 | 39933111 |
| chrX | *BCOR* | 39933101 | 39933284 |
| chrX | *BCOR* | 39933274 | 39933487 |
| chrX | *BCOR* | 39933477 | 39933695 |
| chrX | *BCOR* | 39933685 | 39933912 |
| chrX | *BCOR* | 39933902 | 39934057 |
| chrX | *BCOR* | 39934056 | 39934232 |
| chrX | *BCOR* | 39934222 | 39934433 |
| chr4 | *PDGFRA* | 55140978 | 55141171 |
| chr4 | *PDGFRA* | 55144028 | 55144209 |
| chr4 | *PDGFRA* | 55151970 | 55152169 |
| chr4 | *KIT* | 55589691 | 55589906 |
| chr4 | *KIT* | 55592021 | 55592244 |
| chr4 | *KIT* | 55593533 | 55593758 |
| chr4 | *KIT* | 55594136 | 55594350 |
| chr4 | *KIT* | 55599232 | 55599363 |
| chr15 | *IDH2* | 90631752 | 90631976 |
| chr4 | *TET2* | 106154900 | 106155124 |
| chr4 | *TET2* | 106155114 | 106155255 |
| chr4 | *TET2* | 106155245 | 106155450 |
| chr4 | *TET2* | 106155440 | 106155658 |
| chr4 | *TET2* | 106155648 | 106155853 |
| chr4 | *TET2* | 106155843 | 106156042 |
| chr4 | *TET2* | 106156032 | 106156254 |
| chr4 | *TET2* | 106156244 | 106156445 |
| chr4 | *TET2* | 106156435 | 106156651 |
| chr4 | *TET2* | 106156641 | 106156862 |
| chr4 | *TET2* | 106156852 | 106157077 |
| chr4 | *TET2* | 106157067 | 106157290 |
| chr4 | *TET2* | 106157279 | 106157503 |
| chr4 | *TET2* | 106157493 | 106157707 |
| chr4 | *TET2* | 106157697 | 106157926 |
| chr4 | *TET2* | 106157914 | 106158107 |
| chr4 | *TET2* | 106158097 | 106158311 |
| chr4 | *TET2* | 106158301 | 106158508 |
| chr4 | *TET2* | 106196185 | 106196384 |
| chr4 | *TET2* | 106196374 | 106196596 |
| chr4 | *TET2* | 106196585 | 106196796 |
| chr4 | *TET2* | 106196786 | 106197009 |
| chr4 | *TET2* | 106196999 | 106197224 |
| chr4 | *TET2* | 106197214 | 106197382 |
| chr4 | *TET2* | 106197372 | 106197476 |
| chr4 | *TET2* | 106197466 | 106197684 |
| chr1 | *NRAS* | 115252147 | 115252339 |
| chr1 | *NRAS* | 115256452 | 115256669 |
| chr1 | *NRAS* | 115258642 | 115258862 |
| chr11 | *KMT2A* | 118307508 | 118307730 |
| chr11 | *KMT2A* | 118339410 | 118339601 |
| chr11 | *KMT2A* | 118342301 | 118342521 |
| chr11 | *KMT2A* | 118342511 | 118342729 |
| chr11 | *KMT2A* | 118342806 | 118343033 |
| chr11 | *KMT2A* | 118342986 | 118343209 |
| chr11 | *KMT2A* | 118343256 | 118343480 |
| chr11 | *KMT2A* | 118343876 | 118344086 |
| chr11 | *KMT2A* | 118344128 | 118344348 |
| chr11 | *KMT2A* | 118344416 | 118344631 |
| chr11 | *KMT2A* | 118344618 | 118344844 |
| chr11 | *KMT2A* | 118344788 | 118344974 |
| chr11 | *KMT2A* | 118344901 | 118345117 |
| chr11 | *KMT2A* | 118347468 | 118347688 |
| chr11 | *KMT2A* | 118348744 | 118348963 |
| chr11 | *KMT2A* | 118350829 | 118351048 |
| chr11 | *KMT2A* | 118352495 | 118352723 |
| chr11 | *KMT2A* | 118352684 | 118352908 |
| chr11 | *KMT2A* | 118353099 | 118353317 |
| chr11 | *KMT2A* | 118354860 | 118355079 |
| chr11 | *KMT2A* | 118355570 | 118355756 |
| chr11 | *KMT2A* | 118359272 | 118359490 |
| chr11 | *KMT2A* | 118361806 | 118362026 |
| chr11 | *KMT2A* | 118362451 | 118362671 |
| chr11 | *KMT2A* | 118363738 | 118363951 |
| chr11 | *KMT2A* | 118366949 | 118367154 |
| chr11 | *KMT2A* | 118368602 | 118368825 |
| chr11 | *KMT2A* | 118369981 | 118370202 |
| chr11 | *KMT2A* | 118370532 | 118370703 |
| chr11 | *KMT2A* | 118371656 | 118371873 |
| chr11 | *KMT2A* | 118372413 | 118372629 |
| chr11 | *KMT2A* | 118373060 | 118373282 |
| chr11 | *KMT2A* | 118373329 | 118373551 |
| chr11 | *KMT2A* | 118373497 | 118373721 |
| chr11 | *KMT2A* | 118373897 | 118374121 |
| chr11 | *KMT2A* | 118374045 | 118374272 |
| chr11 | *KMT2A* | 118374363 | 118374586 |
| chr11 | *KMT2A* | 118374571 | 118374799 |
| chr11 | *KMT2A* | 118374774 | 118374999 |
| chr11 | *KMT2A* | 118374954 | 118375177 |
| chr11 | *KMT2A* | 118375163 | 118375390 |
| chr11 | *KMT2A* | 118375380 | 118375607 |
| chr11 | *KMT2A* | 118375670 | 118375895 |
| chr11 | *KMT2A* | 118375891 | 118376116 |
| chr11 | *KMT2A* | 118376084 | 118376310 |
| chr11 | *KMT2A* | 118376376 | 118376581 |
| chr11 | *KMT2A* | 118376640 | 118376861 |
| chr11 | *KMT2A* | 118376882 | 118377107 |
| chr11 | *KMT2A* | 118377071 | 118377299 |
| chr11 | *KMT2A* | 118378136 | 118378356 |
| chr11 | *KMT2A* | 118379754 | 118379963 |
| chr11 | *KMT2A* | 118380620 | 118380843 |
| chr11 | *KMT2A* | 118382567 | 118382759 |
| chr11 | *KMT2A* | 118390297 | 118390527 |
| chr11 | *KMT2A* | 118390582 | 118390808 |
| chr11 | *KMT2A* | 118391431 | 118391644 |
| chr11 | *KMT2A* | 118391950 | 118392174 |
| chr11 | *KMT2A* | 118392524 | 118392745 |
| chr11 | *KMT2A* | 118392775 | 118393002 |
| chr3 | *GATA2* | 128199857 | 128200076 |
| chr3 | *GATA2* | 128200055 | 128200175 |
| chr3 | *GATA2* | 128200582 | 128200725 |
| chr3 | *GATA2* | 128200683 | 128200879 |
| chr3 | *GATA2* | 128202522 | 128202721 |
| chr3 | *GATA2* | 128202711 | 128202879 |
| chr3 | *GATA2* | 128204458 | 128204646 |
| chr3 | *GATA2* | 128204578 | 128204788 |
| chr3 | *GATA2* | 128204779 | 128204922 |
| chr3 | *GATA2* | 128204912 | 128205058 |
| chr3 | *GATA2* | 128205033 | 128205216 |
| chr3 | *GATA2* | 128205543 | 128205771 |
| chr3 | *GATA2* | 128205739 | 128205968 |
| chrX | *PHF6* | 133511598 | 133511809 |
| chrX | *PHF6* | 133511939 | 133512072 |
| chrX | *PHF6* | 133512062 | 133512262 |
| chrX | *PHF6* | 133527347 | 133527553 |
| chrX | *PHF6* | 133527543 | 133527709 |
| chrX | *PHF6* | 133527855 | 133528004 |
| chrX | *PHF6* | 133547404 | 133547596 |
| chrX | *PHF6* | 133547586 | 133547696 |
| chrX | *PHF6* | 133547806 | 133548001 |
| chrX | *PHF6* | 133548854 | 133549067 |
| chrX | *PHF6* | 133549056 | 133549120 |
| chrX | *PHF6* | 133551169 | 133551357 |
| chrX | *PHF6* | 133559196 | 133559416 |
| chr7 | *EZH2* | 148506335 | 148506550 |
| chr7 | *EZH2* | 148507353 | 148507579 |
| chr7 | *EZH2* | 148523390 | 148523575 |
| chr7 | *EZH2* | 148523565 | 148523778 |
| chr5 | *NPM1* | 170837497 | 170837642 |
| chr2 | *IDH1* | 209112993 | 209113209 |
